# Three-Dimensional Vertebral Wedging in Mild and Moderate Adolescent Idiopathic Scoliosis

**DOI:** 10.1371/journal.pone.0071504

**Published:** 2013-08-15

**Authors:** Sophie-Anne Scherrer, Mickaël Begon, Alberto Leardini, Christine Coillard, Charles-Hilaire Rivard, Paul Allard

**Affiliations:** 1 Laboratoire d’ingénierie du mouvement, Department of Kinesiology, University of Montreal, Montreal, Quebec, Canada; 2 Movement analysis laboratory, Istituto Ortopedico Rizzoli, Bologna, Bologna, Italy; 3 Faculty of Medicine, University of Montreal, Montreal, Quebec, Canada; 4 Department of Kinesiology, University of Montreal, Montreal, Quebec, Canada; 5 Human Movement Laboratory, Research Centre, Sainte-Justine Hospital, Montreal, Quebec, Canada; Van Andel Institute, United States of America

## Abstract

**Background:**

Vertebral wedging is associated with spinal deformity progression in adolescent idiopathic scoliosis. Reporting frontal and sagittal wedging separately could be misleading since these are projected values of a single three-dimensional deformation of the vertebral body. The objectives of this study were to determine if three-dimensional vertebral body wedging is present in mild scoliosis and if there are a preferential vertebral level, position and plane of deformation with increasing scoliotic severity.

**Methodology:**

Twenty-seven adolescent idiopathic scoliotic girls with mild to moderate Cobb angles (10° to 50°) participated in this study. All subjects had at least one set of bi-planar radiographs taken with the EOS® X-ray imaging system prior to any treatment. Subjects were divided into two groups, separating the mild (under 20°) from the moderate (20° and over) spinal scoliotic deformities. Wedging was calculated in three different geometric planes with respect to the smallest edge of the vertebral body.

**Results:**

Factorial analyses of variance revealed a main effect for the scoliosis severity but no main effect of vertebral Levels (apex and each of the three vertebrae above and below it) (F = 1.78, p = 0.101). Main effects of vertebral Positions (apex and above or below it) (F = 4.20, p = 0.015) and wedging Planes (F = 34.36, p<0.001) were also noted. Post-hoc analysis demonstrated a greater wedging in the inferior group of vertebrae (3.6°) than the superior group (2.9°, p = 0.019) and a significantly greater wedging (p≤0.03) along the sagittal plane (4.3°).

**Conclusions:**

Vertebral wedging was present in mild scoliosis and increased as the scoliosis progressed. The greater wedging of the inferior group of vertebrae could be important in estimating the most distal vertebral segment to be restrained by bracing or to be fused in surgery. Largest vertebral body wedging values obtained in the sagittal plane support the claim that scoliosis could be initiated through a hypokyphosis.

## Introduction

Scoliosis is a three-dimensional (3D) deformity of the spine with curvatures occurring in all three planes [1]. In adolescent idiopathic scoliosis, the scoliotic deviation has been associated with wedging of both vertebrae and discs. This could be of importance since vertebral body deformation increases as the scoliosis progresses [2], [3]. Furthermore it is believed that vertebral axial rotation in scoliosis can occur with progressive wedging [4]. Several studies have described vertebral wedging [5]–[9] but most of them are based on measurements taken on the frontal plane by means of posterior-anterior spinal radiographs. Reporting separately frontal and sagittal wedging could be misleading since these are projected values of a single 3D morphology deformation of the vertebral body and the maximum scoliotic deviation lies in a plane oblique to the frontal plane [10].

There are however a few studies on 3D vertebral morphology. Masharawi et al. [11] used a 3D digitizer to measure lengths, heights and widths, and their corresponding ratios of dissected thoracic and lumbar vertebrae from 240 non-scoliotic adults. Their findings indicate that vertebral wedging is already present in both the frontal and sagittal planes in the absence of scoliosis and that it is more frequent in women than in men. According to Parent et al. [4], few studies addressed vertebrae morphology in scoliosis and these are often based on vertebral reconstructions obtained from CT-scan [12]. Parent et al. [4] digitized 471 vertebrae of 30 scoliotic and sex-matched non-scoliotic specimens using two approaches. Firstly they defined vertebral wedging by the angle sustained by a least-square plane calculated for each endplate and projected in the frontal and sagittal planes as well as in the plane of the maximal deformity. Their second method of vertebral wedging is based on vertebral heights taken at the periphery of the vertebral body. Though the former technique is more practical when digitizing cadaveric specimens, the latter method can be easily applied in a clinical setting where bi-planar radiographs are taken routinely.

Frontal plane wedging has been well documented for scoliotic patients with severe deformity. In Modi et al. [7] the average Cobb angle was approximately 27° and reached 53.1° in Wang et al. [9]. In another study [13] where the severity of the deformity was not available, 21 of the 30 scoliotic subjects were male and the overall mean age was 44.2 years implying patients with a severe scoliosis. Some studies included boys and girls [5] as well as right and left scoliosis [8] while others had different curve types [7], [8], [14]. To the best of our knowledge there is no clinical study on vertebral wedging comprising solely of mild (less than 20°Cobb angle) and moderate scoliotic girls prior to any form of treatment which could document the initial stages of scoliosis progression.

In this study we have combined the approaches described by Parent et al. [4], [13] and Stokes and Aronsson [8] to estimate the overall 3D wedging, which was calculated in three different geometric planes with respect to the smallest edge of the vertebral body. The first objective of this study on 3D vertebral body wedging is to determine whether this wedging is present in mild scoliosis and whether it increases in moderately deformed spine of untreated adolescent idiopathic scoliosis girls. To test if there was a preferential vertebral level, wedging values were calculated from the third vertebrae above to the third below the apex of the spinal curve in the two groups of scoliotic severity. The effect of vertebral positions on wedging was also verified. This was to test the hypothesis that the vertebrae below the apex are more deformed according to the Hueter-Volkmann Law [15]. Finally, we wished to determine if there was a preferred plane of deformation with increasing scoliotic severity.

## Methods

Twenty-seven adolescent idiopathic scoliotic girls having an average age of 13.1±1.7 years and diagnosed by an orthopedic surgeon according to the criteria given by Bunnel [16] participated to this study. None was under active treatment whatsoever. Subjects were excluded from the study if they were wearing a foot orthosis; had a limb length discrepancy of more than 1 cm and had any signs of orthopedic or neurological anomalies. All scoliotic girls had a set of postero-anterior and lateral radiographs taken at their initial visit and for 13 of them we have included a second set of radiographs obtained prior to bracing. The latter radiographs were arbitrary considered as independent measures because the average time interval between radiographs was 12.3 months and the average progression of untreated scoliosis of 5°. In all, 40 radiographs were considered for the analysis. The average Cobb angle was 21.6° ±9.1° and ranged between 10° and 50°. There were 17 right and 6 left thoracic curves and 5 right and 12 left thoraco-lumbar curves. All measurement procedures were explained to each subject and a written informed consent approved by the ethics committee of Sainte-Justine hospital was obtained from each subject and their parents or guardians prior to testing. The research ethics committee of Sainte-Justine hospital also approved the study.

The girls were then divided into two groups to separate the mild from the moderate spinal scoliotic deformities. The median Cobb angle was 20° and it was selected as the division point between these two groups. Such a distribution was used in previous studies [17], [18] and closely corresponds to the standard demarcation between mild and moderate scoliotic curves [16], [19]–[22]. The mean demographic characteristics of the untreated mild and moderate scoliotic groups are given in Table 1.

**Table 1:** Mean age and Cobb angle values with standard deviations, and Cobb angle range given for the mild and moderately severe scoliosis groups.

Group	n	Age (years)	Mean Cobb angle (degrees)	Cobb angle range (degrees)
Mild scoliosis	20	12.5±1.5	14.9±3.1	10–19
Moderate scoliosis	20	13.6±1.7	28.2±8.1	20–50

Forty standardized bi-planar radiographs were taken with the EOS® X-ray imaging system based on the Nobel Prize work of Georges Charpak. This system allows for full-body weight-bearing standing orthogonal set of digital radiographs. These are taken simultaneously to ensure that the same spine configuration is projected onto the postero-anterior and sagittal radiographs simultaneously. The EOS® system has been developed for orthopedic applications especially for assessing spinal deformities [23]–[25].

**Figure 1:**
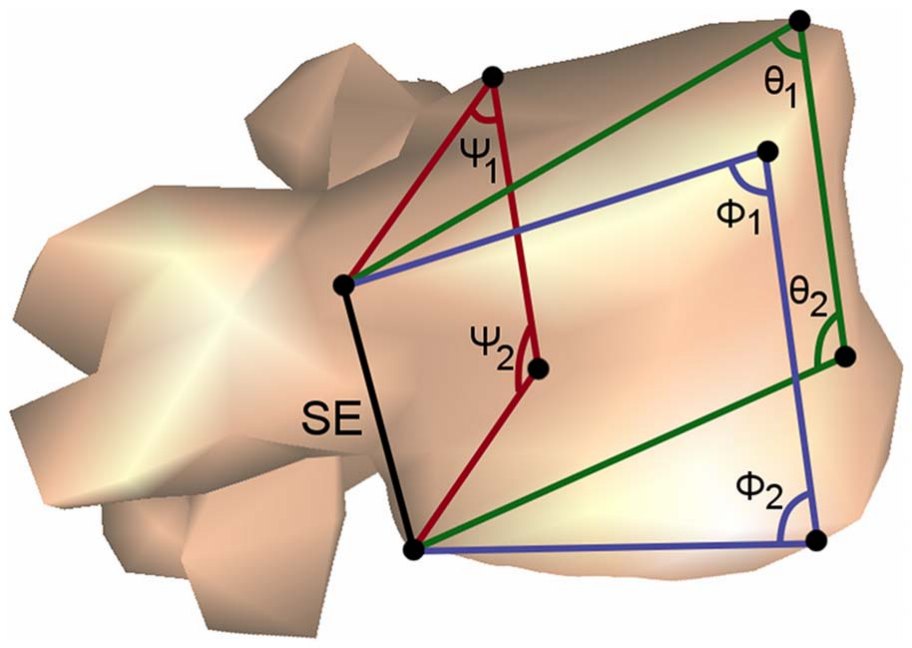
Selected landmarks and measured angles on each thoracic and lumbar vertebrae. Points represent the eight vertebral body corners as seen on the radiographs. The spatial angles were measured with respect to the smallest edge (SE) representing the frontal, sagittal and diagonal planes wedging. Ψ, φ and θ represent respectively the postero-frontal, sagittal and diagonal angles with 1 and 2 indicating the superior and inferior angles. Each superior and inferior angles of a plane were summated to characterize vertebral wedging.

**Figure 2:**
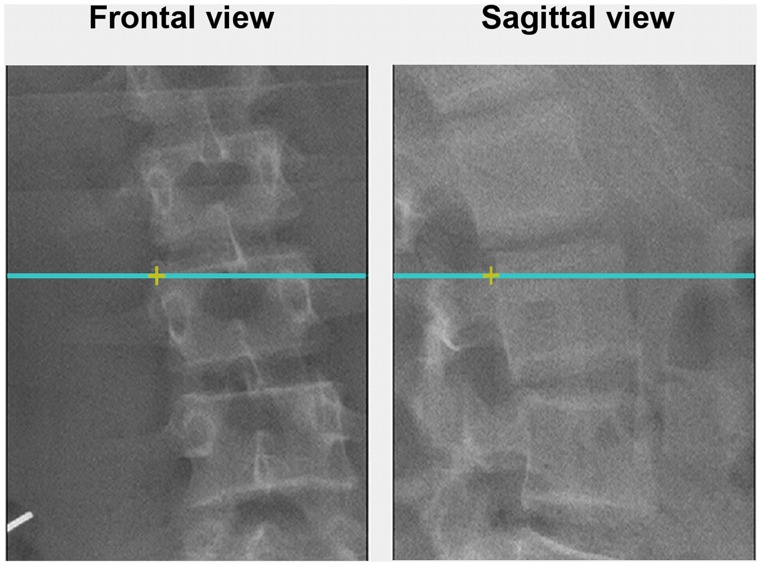
Thoracic and lumbar vertebrae digitization. Firstly a bony landmark was identified on the postero-anterior radiograph. A horizontal line drawn from this point was projected on the lateral radiograph. The intersection between the horizontal line and the edge of the vertebral body ensured that the same bony landmark was identified in each pair of radiographs.

Each radiograph was manually digitized to obtain the planar coordinates of 8 bony landmarks on each of the 12 thoracic and 5 lumbar vertebrae. These points correspond to the vertebral body corners [26] as shown in Fig. 1, and enable the calculation of vertebral wedging in several planes compared to the capability of the EOS software, which only uses four bony landmarks by vertebrae [27]. First a bony landmark was identified on the postero-anterior radiograph. A horizontal line drawn from this point was projected on the lateral radiograph as illustrated in Fig. 2. The intersection between the horizontal line and the edge of the vertebral body ensured that the same bony landmark was identified in each pair of radiographs. The 3D position of the bony landmarks was then reconstructed from planar coordinates from both planar radiographs following a calibration technique. Though EOS® stereo-radiographs are calibrated when processed by its associated software, visualisation of these images by independent software requires computational modifications. The procedure is described in part by Allard et al. [28], [29] and has been adapted for the EOS® system. This adjusted procedure was tested on 26 fiducial landmarks located in known positions within a calibration object measuring 140 mm by 210 mm by 410 mm. The average positional error was 1.35±0.79 mm and the angular error was 0.1±1.0°, which compare well with those reported by Ills and Somoskey [30] for the EOS® reconstruction software system. Since most wedging angles ranged between 3° and 5°, our average angular error of 0.1° should have little impact on the interpretations of the results.

When a second compensatory curve was present, the largest between the two was selected for the analysis. The apical or apex vertebra and three vertebrae above and three below it were analyzed. Wedging was measured by using the 3D coordinates of the eight points reconstructed from the bi-planar radiographs. Since the vertebral body is not necessarily a perfect cylinder of constant height, its shortest vertical edge was identified first as shown in Fig. 1. It was found on the posterior face of the vertebral body and on the concave side of the scoliotic curve. Then the spatial or 3D angle sustained by the upper line taken between the uppermost corner of the smallest edge to an opposite corner and its lateral edge was calculated. A corresponding lower spatial angle was measured similarly but between the lines joining the lowermost corners. To characterize the 3D vertebral body geometry these two spatial angles were summated. In a cube with parallel edges this sum is 180° representing no wedging at all. For any hexahedron (deformed cube) the sum would be different. To express vertebral wedging with respect to an un-deformed cube, 180° was subtracted from the sum of the two angles. This method to calculate vertebral wedging is similar to that of Stokes and Aronsson [8], where a line was drawn across the superior and inferior endplates of each vertebra on a frontal plane radiograph and to that of Parent et al. [13], where a three-dimensional plane was fitted for each vertebral endplates of their cadaveric specimens.

Wedging was calculated with respect to the three other vertebral body edges as shown in Fig. 1. For this reason postero-frontal vertebral wedging was defined with respect to the other posterior lateral edge. Sagittal vertebral wedging was measured with respect to the anterior edge located on the same side of the smallest edge. Finally the diagonal vertebral wedging was in relation to the edge located diagonally in front of the smallest one.

A factorial analysis of variance was performed to determine the effect of the scoliosis severity on seven vertebral Levels (apex and each of the three vertebrae above and below the apex). A second factorial analysis of variance was performed to determine the effect of the scoliosis severity on three Positions (3 superior vertebrae, apex vertebra and 3 inferior vertebrae) and three wedging Planes (postero-frontal, sagittal and diagonal). A p value equal or less than 0.05 was considered as statistically significant. A Bonferroni post-hoc analysis was performed if a statistically significant difference was found.

Data related to this study was not made publicly available as it would breach confidentiality related to human subject research and that all data must be destroyed after a given number of years by the principal investigator.

## Results

**Figure 3:**
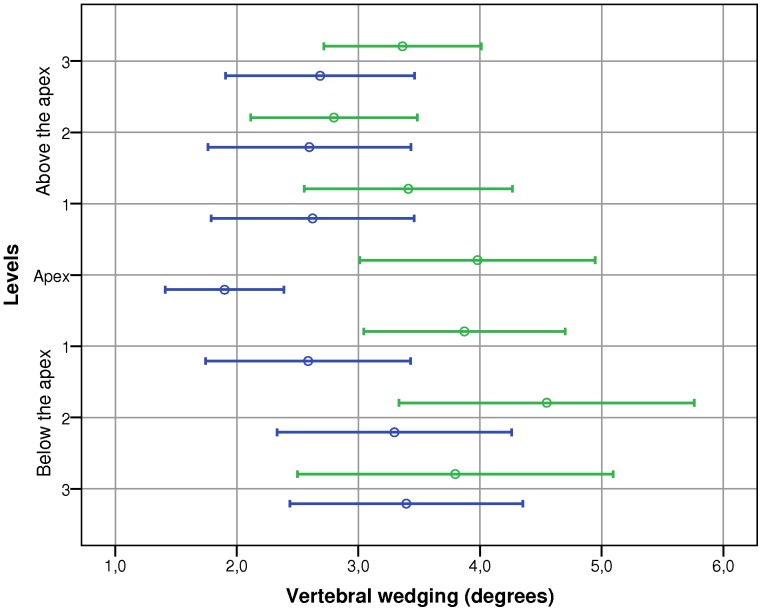
Extent of wedging at the apex and at each of three vertebrae above and below. Circles represent the average wedging and bars represent the 95% confidence interval. The mild scoliosis group is in blue and the moderate scoliosis group is in green.

The extent of wedging at the apex and at each of three vertebrae above and below it for the mild and moderate scoliosis groups is shown in Fig. 3. The analysis presented a main effect of Group (F = 15.99, p<0.001), but not of vertebral Levels (F = 1.78, p = 0.101) and interaction.

**Figure 4:**
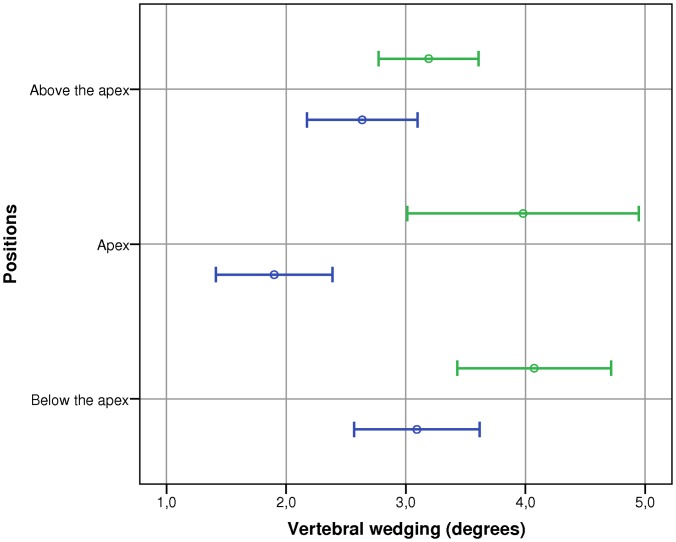
Combined effect of three superior and three inferior vertebrae wedging compared to the apex vertebra. Circles represent the average wedging and bars represent the 95% confidence interval. The mild scoliosis group is in blue and the moderate scoliosis group is in green.

The combined effect of three superior and three inferior vertebrae wedging compared to that at the apex vertebra for the two scoliotic groups are shown in Fig. 4. There was a main effect of Group (F = 21.87, p<0.001) and of Position (F = 4.20, p = 0.015). No significant interaction between the Group and Position was found. Post-hoc analysis demonstrated that the inferior group of vertebrae displayed greater wedging (3.6°) than those of the superior group (2.9°) of vertebrae (p = 0.019). Wedging at the apex vertebra (2.9°) was not statistically different from those of the superior or inferior groups of vertebrae.

**Figure 5:**
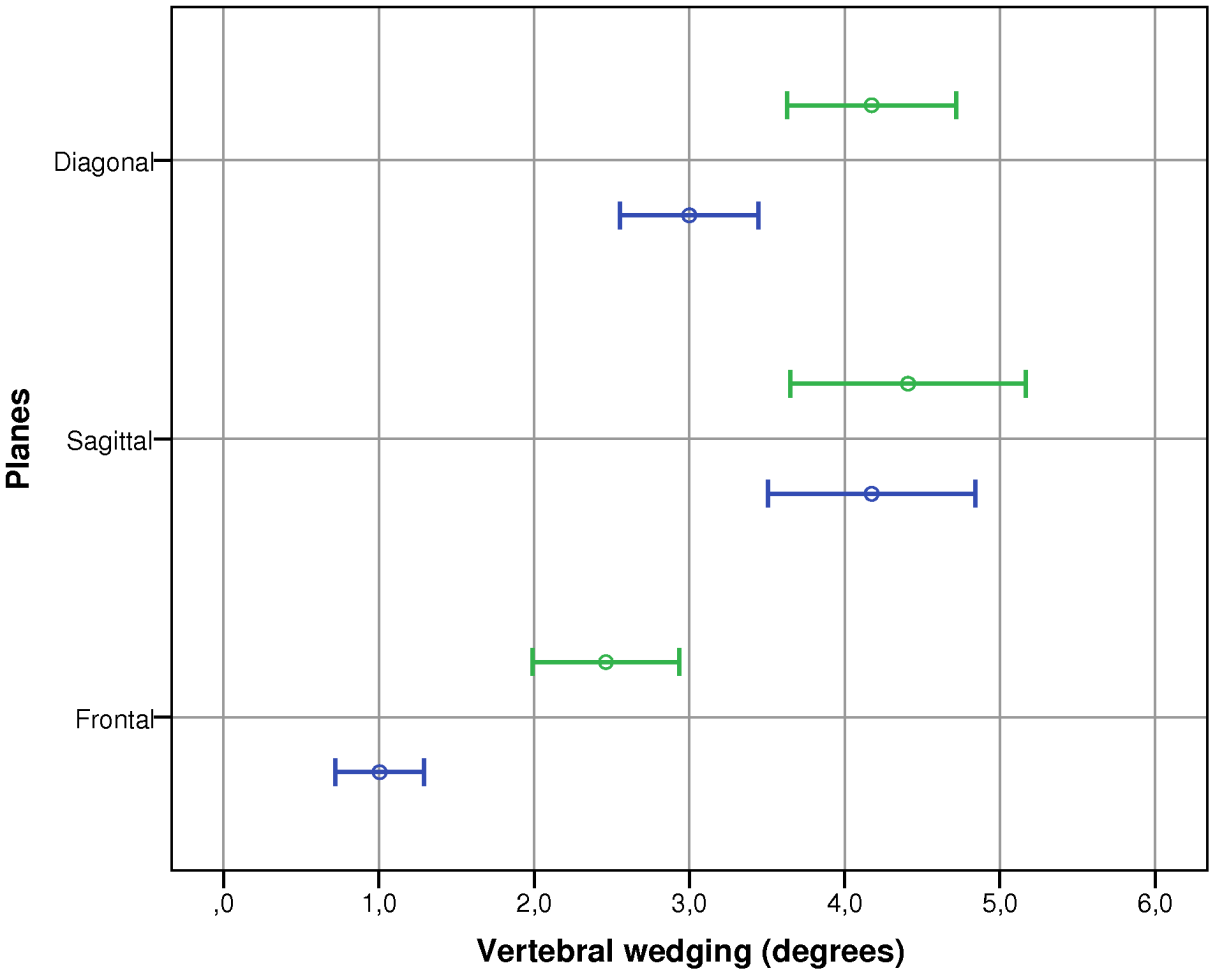
Vertebral wedging values obtained for the frontal, sagittal and diagonal edges. Circles represent the average wedging and bars represent the 95% confidence interval. The mild scoliosis group is in blue and the moderate scoliosis group is in green.

The final results were to determine if there was a preferential plane of vertebral wedging. Figure 5 illustrates vertebral wedging values obtained for the postero-frontal, sagittal and diagonal edges. There was a main effect of Plane (F = 34.36, p<0.001). No significant interactions between Group and Plane were found. Post-hoc analysis revealed that wedging was significantly (p≤0.03) the greatest along the sagittal plane (4.3°) followed by the diagonal (3.6°) and postero-frontal (1.7°) planes.

## Discussion

According to Wang et al. [9] and Stokes and Aronsson [8], disc and vertebral body wedging could play an important role in the progression of adolescent idiopathic scoliosis since it usually increases during the rapid growth period in the adolescence [31], [32]. There is a dearth of studies on 3D vertebral wedging and these are limited by cadaver-based measurements whereas nearly all the clinical studies used mostly postero-frontal plane radiography measurements. Additionally, wedging values were obtained from mixed genders in the scoliotic groups and by severity and type of spinal curves. This makes the interpretation of research findings difficult. To the best of our knowledge the present clinical study is the only one using 3D wedging measurements in girls with mild and moderate scoliosis.

Stokes and Aronsson [8] did not find an increase in the amount of vertebral and disc wedging between initial and follow-up radiographs of the scoliotic patients. This can be explained in part by a large initial Cobb angle and a Cobb angle larger than 15° in the first radiograph of 22 patients out of 27 representing a moderately severe scoliotic group. An important finding of our study is that vertebral wedging is present even in girls with a mild thoracic or thoraco-lumbar scoliosis (average Cobb angle of 14.6°). This is not too surprising since Marsharawi et al. [11] reported vertebral wedging in non-scoliotic individuals and that it was found larger in women than in men. Similarly to our findings, Xiong et al. [33] reported vertebral wedging in small scoliotic deformities (4° to 7° Cobb angle). Vertebral wedging disturbance in the early pathomechanism of scoliosis is not an isolated phenomenon. Asymmetrical growth of the pelvis [17], [18], poorer postural control and sensory deficit in scoliotic patients with a Cobb angle greater or equal to 15° were reported by Haumont et al. [34] and Simoneau et al. [35]. We do not have sufficient information to ascertain that the observed wedging in the mild scoliotic group is directly related to the spinal deformity on-set and progression or that it is part of the natural growth and development of the vertebrae. Abnormal vertebral wedging in girls with mild scoliosis remains an open question.

Vertebral wedging has been reported to be the largest at the apex vertebra and to become smaller as the inferior and superior vertebrae are more distant to the apex [5], [7], [8], [14]. These observations were essentially based on postero-frontal plane measurements of the angle sustained between the lines drawn across the superior and inferior endplates of each vertebra in the curve. Notwithstanding the intrinsic limitations associated with frontal plane radiographic measurements, most of the subjects had advanced if not severe scoliosis. Parent et al. [4] also reported similar findings in scoliotic specimens but where the Cobb angles were not available and the subjects mean age was above 40 years old. In our investigation vertebral wedging at the apex was not different from that of the adjacent inferior and superior vertebrae. This could be representative of mild and moderately severe scoliosis. This is in agreement with Will et al. [14]: from a longitudinal study of 150 adolescent idiopathic scoliosis patients, they concluded that the Cobb angle initially increased through disc wedging rather than vertebral wedging during the rapid growth spurt. Our results do not preclude that maximum wedging could occur at the apex vertebra in older patients with severe scoliosis.

Our method of calculating vertebral wedging differs from others because it involves bi-planar radiography and 3D calculations as enabled by the modern EOS® system. By using the projection of the endplates on a postero-anterior radiograph, the orientation of the lateral edges of the vertebra is excluded. With 3D measurements, the endplates and vertebral edges are not considered horizontal or vertical. In other words the vertebral body is not considered as a slanted but rather a deformed cube. In this method, wedging is calculated in a reference system specific to each vertebra. A future study involving the calculation of wedging in the plane of maximal deformity could bring additional information on the influence of each vertebra on the scoliotic deviation.

The progression of vertebral wedging could be attributed to an abnormal vertebral growth in the skeletally immature spine of scoliotic patients [4], [7], [9], [36]. This bone growth-rate dependence on stress magnitudes assumption is based on the Hueter-Volkmann Law [15]. In our study, wedging was significantly more pronounced in the inferior three vertebrae to the apex. This group of vertebrae bears the greatest endplate pressure, retarding bone growth (Hueter), while those at the apex and above would withstand lesser pressure, accelerating growth (Volkmann). This possible vicious cycle characterized by concomitant asymmetrical spinal loading and growth could lead to the progression of the spinal deformity as hypothesized by Stokes [37], [38] and that it could occur in the early on-set of scoliosis.

Wedging in the sagittal plane was reported by Parent et al. [4] to be of lesser importance than that in the frontal plane in a morphometric analysis of scoliotic specimens. They concluded that their results do not support Dickson [39] assertion that scoliosis stems from a modification of the sagittal profile buckling of the spine due to a progressive hypokyphosis followed by lordosis. In our study, postero-frontal plane wedging values increased with the progression of the spinal deformity but those values obtained in the sagittal plane were the largest. This observation not only supports Dickson [39] but is consistent with Marsharawi et al. [11], Holt et al., 40 and Milne and Williamson 41. However, randomized controlled trials could prove more definitive evidence.

Vertebral axial rotation could affect the apparent wedging as seen from the posterior-anterior radiograph. In such a case the lateral plane wedging is in part projected on the frontal plane. According to Stokes and Aronsson [8] the projection would have little effect in their 2D study. The magnitude of sagittal plane wedging at the curve apex was considered relatively smaller than that of the frontal plane wedging and that it would take a substantial vertebral rotation to make sagittal plane wedging completely visible. Notwithstanding that our study is based on 3D morphologic reconstruction of vertebral bodies, the influence of vertebral rotation was minimized by identifying simultaneously the similar anatomical landmarks on both radiographs. Furthermore, wedging was considered not only as the inclination of the vertebral endplates but also as the total body deformation by considering the vertebral body as a hexahedron.

Though no definite conclusions can be drawn about the causal relationship between the vertebral wedging and spinal curve progression in adolescent idiopathic scoliosis, the present study brings new insight in the development of scoliosis from its onset. Few studies have reported quantitative measurements of vertebral wedging in girls with mild scoliosis. The progression of vertebral wedging from the early on-set observed in girls with a mild scoliosis could lead to better corrective spinal surgical strategies and more efficient corrections [4]. Another important finding of this study was that wedging is the largest in the three inferior vertebrae to the apex of the spinal curve. This could have a clinical implication when deciding the number of vertebrae to instrument below the apex during spinal surgery correction. The aim is to preserve motion at the distal segment with respect to the instrumented lower end vertebra [42] while allowing a greater distribution of the functional motion across more vertebral levels [43]. However, a short fusion of vertebrae could lead to an unstable correction and trunk imbalance [44].

### Conclusion

The main findings of this 3D measurements study are that vertebral body wedging is present in girls with a mild scoliosis of less than 20° and that it progresses. Wedging was more pronounced in the three vertebral bodies immediately located below the apex. This could be an important consideration in estimating the most distal vertebral segment to be restrained by bracing or to be fused in surgery. Though postero-frontal plane wedging values increased with the progression of the spinal deformity, values obtained in the sagittal plane were the largest. This supports the claim of Dickson [39] that scoliosis could be initiated through a hypokyphosis.
